# Heavy Metals Ions Removal from Local Tarnita Aquatic Streams by Reusable Zwitterionic Acrylic Ion Exchange Resins

**DOI:** 10.3390/polym17233173

**Published:** 2025-11-28

**Authors:** Marcela Mihai, Alina-Petronela Moraru, Ramona Ciobanu, Florin Bucatariu, Marius-Mihai Zaharia

**Affiliations:** Petru Poni Institute of Macromolecular Chemistry, 41A Grigore Ghica Voda Alley, 700487 Iasi, Romania; marcela.mihai@icmpp.ro (M.M.); moraru.alina@icmpp.ro (A.-P.M.); ramona.ciobanu@student.tuiasi.ro (R.C.); fbucatariu@icmpp.ro (F.B.)

**Keywords:** ionic exchange resins, cross-linking, heavy metal ions sorption, polluted river water, wheat germination

## Abstract

This study represents comprehensive research that arises from the advanced sorption properties of zwitterionic resin beads, which were tested on simulated mono- and multicomponent heavy metal ion (HMI)-polluted water, compared to the stream collected in the Tarnita mine area. Ionic exchange resins (IExRs) were first synthesized in cationic form from a highly crosslinked (8%) acrylic copolymer, by introducing different side groups containing amino functionalities, such as ethylenediamine, triethylenetetramine, and hydrazine hydrate. The corresponding zwitterionic form of each IExR was obtained by reacting the cationic resins with sodium chloroacetate. The structures and morphologies of the synthesized resins were characterized using scanning electron microscopy and infrared spectroscopy. Successful removal of Cu(II), Fe(II), and Mn(II) was quantified by using atomic absorption spectroscopy. Tests with multicomponent synthetic solutions revealed the following typical order of retention: Cu(II) > Fe(II) > Mn(II). In the case of water samples collected from the Tarnita area, the zwitterionic resins were able to retain approximately 93.8% Mn(II), 94.7% Fe(II), and >95.5% Cu(II); in all instances, the concentration of Fe(II) was significantly higher than that of Cu(II) and Mn(II). Additionally, sorption isotherms, kinetics, and thermodynamic parameters were studied. Wheat germination was included to test the efficiency of the batch sorption using IExRs, compared to the stream collected from Tarnita, highlighting how the water cleaning process leads to healthy plant growth. The results demonstrate that, after IExRs sorption the tested HMIs content is below the permissible maximum level for surface water, effectively mitigating the pollution of the steam near to the Tarnita closed mine area, removing the main contaminants found in it.

## 1. Introduction

Over the last years, heavy metal ions (HMIs) have been accumulating in the environment due to a range of industrial development and expansion processes, including mining, metallurgy, and chemicals [1,2]. Unlike organic pollutants, HMIs cannot biodegrade, thus remaining in an ecosystem for decades and causing irreversible impacts on the environment [3,4,5]. Once in the environment, HMIs accumulate in soils and water, are taken up by living organisms, undergo biomagnification in the food web, and ultimately pose substantial risks to both the environment and human health [6,7]. This problem can be exacerbated in abandoned mining sites, where improperly constructed ponds continue to release harmful materials into the adjacent ecosystems [8,9,10]. As a previously mined barite area, the closed Tarnița mine has become a significant environmental concern [11]. After the barite mine closed, the area around it remained contaminated, because pollution does not stop with the cessation of the mining processes [12]. The ongoing release of HMIs into nearby soil and surface water, caused by a lack of control and cleanup measures, creates unsafe conditions, affecting the community, fauna, and flora in adjacent ecosystems [13,14,15]. Recent onsite investigatory samples have shown a disturbing increase in the pollutant population, indicating the need for a comprehensive remediation which could manage the contaminant footprint of the mining operations and their relationship to the sedimentary HMIs found in the ecosystem [13,14]. For sampling surface water sources in proximity to the Tarnița mining area, HMIs in aquatic ecosystems were lensing the metal-ion content utilizing atomic absorption spectrometry (AAS), as it is both an easy and sensitive method for measuring contaminant levels. The analytical data reported hazardous concentrations of several contextually important HMIs, including manganese (Mn^2+^) 1.95 mg/L, copper (Cu^2+^) 19.91 mg/L, and iron (Fe^2+^) at 154.59 mg/L [16]. These concentrations are above the recommended limits for surface water established by international environmental agencies [17,18]. The significantly high concentrations of Mn^2+^ and Cu^2+^ suggest that mining contaminants of residual ores and mining-related waste were left uncontrolled or that the treatment equipment was left unattended. The combination of elevated Mn^2+^ and Cu^2+^ levels suggests that uncontained mine waste rock or industrial waste was left in the environment, while raised Fe^2+^ levels indicate that iron-bearing minerals may have leached from the environment as a consequence of acid mine drainage. The combined effects of these contaminants, together with the leaching of pollutants into the Tarnița surface water, highlight the area’s significance as a case study for identifying efficient water filtration solutions. There are in-depth discussions and detailed summaries in the literature regarding traditional methods for the removal of HMIs, where potential options include electrochemical treatments, coagulation–flocculation, membrane filtration, and precipitation [4,19,20]. While all of the methods described above offer certain advantages under specific conditions, several limitations have been well-documented, including high-priced/high operational costs, limited selectivity, substantial energy demands, and the generation of a significant amount of hazardous sludge that requires further treatment and disposal [21]. Consequently, among the cost-effective and sustainable pollutant removal approaches, adsorption and ion exchange processes gain prominence as the way forward [22].

Ion exchange resins (IExRs) are highly selective, flexible, and able to be reused or recycled, which makes them a very attractive material for retaining HMIs from polluted sources of water [23,24]. These synthetic polymers can remove contaminants through a relatively simple method of exchanging the counterions in their structure with certain metal ions from a solution. The chelating IExRs have the advantage of forming stable coordination complexes with metal ions [25,26]. Their performance can be easily tuned by the chemical structure, crosslinking degree, and types of functional groups [27,28]. Among the various synthetic resins for environmental applications, those based on crosslinked acrylic polymers are currently the most promising. Through chemical modification, the materials can be endowed with appropriate physical and chemical characteristics, including hydrophilicity, functionalization with specific chelating groups, and adjustable porosity [29]. Among the functional groups, the amino/imino (–NH_2_/–NH–), carboxyl (–COOH), and amide (–NH–CO) groups have shown great promise for donor–acceptor interactions that selectively bond divalent and trivalent metal cations [30,31]. These functional groups function as Lewis bases or electron pair donors and could create stable metal–ligand complexes by coordinating with electron-deficient metal ions (Lewis’s acids) [32], which are trapped within the resin matrix [33,34]. Although both the chemical structure and sorption behavior of IExRs already been described in the scientific literature, there is still a lack of synergistic evaluations of IExRs synthesis, structure optimization, and practical development—especially relevant for the closed Tarnița mine, where HMIs have contaminated the nearby water. Previous research has largely been based on controlled laboratory batch experiments, usually utilizing synthetic metal ion solutions that do not reasonably reflect the complexity of the polluted water. Even in the limited field application studies that have utilized some IExRs, the laser focus evaluation of how crosslink density affects mechanical stability and resin sorption performance has not been thoroughly evaluated.

This study systematically investigated the synthesis, physicochemical properties, and functional applications of new IExRs based on 8% crosslinking density acrylic copolymers. The IExRs were designed to specifically target HMIs usually found in polluted waters in the mining areas (mainly Fe(II), Cu(II), and Mn(II)) by functionalizing them with ethylenediamine and triethylenetetramine or with hydrazine hydrate, yielding weak cationic resins bearing amino groups. In addition, the formation of zwitterionic resins via the reaction of weak cationic resins with sodium chloroacetate was followed. Sorption equilibrium and kinetic tests demonstrated the functional stability of the new IExRs, as well as the effectiveness of the resin’s functional groups. In this study, mono-HMI and multi-HMI synthetic waters, as well as water samples collected from the Tarnița area, were tested by evaluating the IExRs’ sorption capacity and selectivity to provide contextual knowledge regarding the materials’ feasibility and operational performance for extensive clean-up projects. This research aims to support the development of technically practical, economically reasonable, and ecologically preferred designs for HMI removal from contaminated water sources. By correlating advanced polymeric chemistry with specific applications to the environment, this study seeks to provide a scalable solution to water purification in post-mining landscapes and other vulnerable open systems.

## 2. Materials and Methods

### 2.1. Materials

**Scheme 1:**
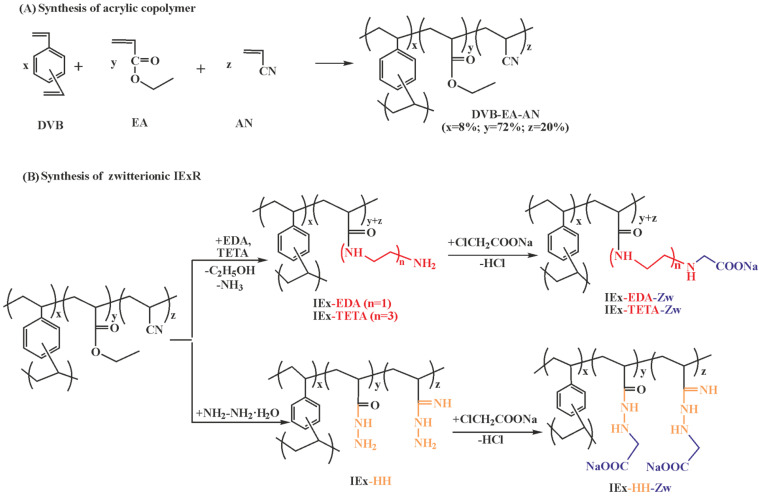
Schematic representation of (**A**) acrylic copolymer synthesis followed by (**B**) cationic and zwitterionic IExRs synthesis.

A copolymer of divinylbenzene (DVB), ethyl acrylate (EA), and acrylonitrile (AN) with an 8% crosslinking degree was first obtained, followed by functionalization in accordance with our previously described methodology [35]. Briefly, ethylenediamine (EDA) or triethylenetetramine (TETA) and hydrazine hydrate (HH) were used to produce weak cationic IExRs. The process of obtaining zwitterionic (Zw) IExRs involved the reaction of weak cationic IExRs with sodium chloroacetate (Scheme 1).

The following compounds were used as received for the synthesis of the copolymer and the IExRs: ethyl acrylate (99.5%, Fluka, Seelze, Germany), acrylonitrile (99%, Fluka), divinylbenzene (99.4%, Fluka), ethylenediamine (98%, Merck, Darmstadt, Germany), triethylenetetramine (TETA; 98%, Fluka), and hydrazine hydrate. Sodium chloroacetate was synthesized via the neutralization of monochloroacetic acid (99.9%, Merck) with sodium hydroxide (95%, Sigma Aldrich, St. Louis, MO, USA) in aqueous media. Salts of CuSO_4_⋅5H_2_O, FeSO_4_⋅7H_2_O, and MnSO_4_⋅H_2_O were acquired from Sigma-Aldrich (~95%, Sigma-Aldrich) and used as purchased. IExR regeneration and reutilization tests were carried out using 1N aqueous solutions of NaOH and HCl (95%, Sigma-Aldrich). For all studies, ultrapure water (0.552 µS·cm^−1^) was used, including solution preparation and washing using an Evoqua Ultra Clear TPTWF.

### 2.2. HMIs Sorption Experiments

To test for HMI sorption, a volume of 1 mL of swelled IExR beads was placed in a beaker, to which 20 mL of polluted water was added (simulated mono-HMI and multi-HMI aqueous samples using Cu(II), Fe(II), and Mn(II) ions, as well as polluted water samples collected from the Tarnita area) and kept under shaking at 250 rpm using the Orbital Shaker-Incubator ES-20 for 24 h if not specified or for different periods of time, at a temperature of 25 ± 1 °C. The appropriate salts were dissolved in ultrapure water to create fresh stock solutions for each HMI. The starting concentration of the mono-HMI and multi-HMI aqueous solutions was 1 mM divalent metal ion (Me(II)), and their pH was corrected to about 5 using 1 N HCl or NaOH for mono-HMI and multi-HMI; for Tarnita water, the pH was not adjusted. The effectiveness of IExR as a sorbent was assessed by regenerating the HMIs-loaded resins for two hours, utilizing a 1 N HCl aqueous solution. Subsequently, the IExR was activated with 1 N NaOH and extensively rinsed with ultrapure water to equilibrate the pH, being further reused in a new HMI sorption cycle.

### 2.3. Characterization Methods

*FTIR-ATR spectroscopy* confirmed the synthesis of the cationic/zwitterionic IExR. The samples FTIR-ATR spectra were obtained using an IR Tracer-100 FT-IR spectrometer (Shimadzu Corporation, Kyoto, Japan) with a GladeATR module (PIKE Technologies, Madison, WI, USA).

A Verios G4 UC Scanning Electron Microscope was used, operating at an accelerating voltage of 5 kV in high vacuum, equipped with a concentric backscattered detector (CBS). To enhance the electrical conductivity, the samples were covered with a layer of 10 nm platinum, utilizing a Leica EM ACE200 Sputter Coater (Leica Microsystems, Wetzlar, Germany). Before characterization, IExR was lyophilized at −57 °C for 48 h using an ALPHA 1–2 LD plus freeze dryer (Martin Christ, Osterode am Harz, Germany). The assessment of the IExR in terms of elemental composition, before and after the carboxymethylation reaction, was conducted using an EDAX Octane Elite Energy Dispersive X-ray analyzer (AMETEK Inc., Mahwah, NJ, USA) attached to a Verios G4 UC Scanning electron microscope (Thermo Fisher Scientific, Waltham, MA, USA).

The volume weight (Wv), given in g·mL^−1^, was obtained by weighing a known initial volume of completely water-saturated resin during drying until a constant load value was achieved.

The volume and weight exchange capacities (Ev, mEq·mL^−1^, and EW, mEq·g^−1^, respectively) specifically refer to the exchange capabilities of weak bases and weak acids [35,36]. In summary, 10 mL of IExRs were treated with 1 N HCl to evaluate their exchange capacity. After removing the excess HCl using a mixture of 1:2 (*v*/*v*) water/methanol, the eluent’s HCl content was determined by titrating it with a standardized 1 N NaOH solution. Each test was performed three times, and the average results—with less than 5% variation—were reported. The two ionic forms of the zwitterionic resins were also evaluated. The volume and weight exchange capacities were determined using the equations presented in Table S1, Supplementary Material.

The diameters (Dm, mm) of the IExRs beads were evaluated by a Morphologi G3SE device (Malvern Instruments Ltd, Malvern, UK). For this, the IExRs were distributed on a glass plate, and the well-formed, non-aggregated resin beads with diameters between 0.3 and 1.5 mm were measured.

The HMIs concentration remaining in the supernatant after IExRs sorption was evaluated using a ContrAA 800 Spectrometer (Analytik Jena GmbH+Co. KG, Jena, Germany) outfitted with a continuous radiation source as a Xenon short-arc lamp. To avoid the interference of a possible formed metal complex, before analysis the solutions were diluted with 0.5 wt.% HNO_3_. The Cu(II), Fe(II), and Mn(II) corresponding peaks absorbance occurred at 324 nm, 248 nm, and 279 nm, respectively. In order to quantify the metals, external calibration curves were constructed using standard solutions of Cu(II), Fe(II), and Zn(II), with concentrations in the range of 0.1 to 5.0 mg/L. The calibration curves showed ‘excellent’ linearity with correlation coefficients (R^2^) ≥ 0.999 for the three metals. The limits of detection (LODs) were determined as three times the standard deviation of the blank, and were found to be 0.02 mg/L for Cu(II), 0.03 mg/L for Fe(II), and 0.01 mg/L for Zn(II); the limits of quantification (LOQs), as ten times the standard deviation of the blank, were found to be 0.06 mg/L for Cu(II), 0.09 mg/L for Fe(II), and 0.03 mg/L for Zn(II). Quality control was provided through the use of certified reference materials and triplicate sample (n = 3) measurements. Recoveries ranged from 95% to 105% and relative standard deviations (RSDs) were less than 5%, showing that the method was both precise and accurate. The measurements were conducted in an air/acetylene flame of 4–8 mm height and a consistent flow rate of 50 L·h^−1^. The equations used to calculate the IExRs sorption capacity (SC) and retention efficiency (RE) are presented in Table S1.

### 2.4. Wheat Germination Experiments

The wheat germination experiments were performed to assess the toxicity of the contaminated water collected from the Tarnita mine area compared to the water after IExRs sorption, employing a previously established methodology [11,37]. In summary, 5 mL of Tarnita-collected water or the resulting supernatants and 50 wheat seedlings were placed in Petri plates at 25 °C for seven days. For all experiments, three replicates were performed and compared with the seedlings of a control sample when using distilled water. After seven days, the dead seeds, germinated seeds, and grown seedlings were counted. The wheat young plantlets (grown wheat seeds) were collected, and their total height (H, cm) and total mass (M, g) were quantified.

## 3. Results

### 3.1. IExRs Structural and Morphological Characterization

**Figure 1:**
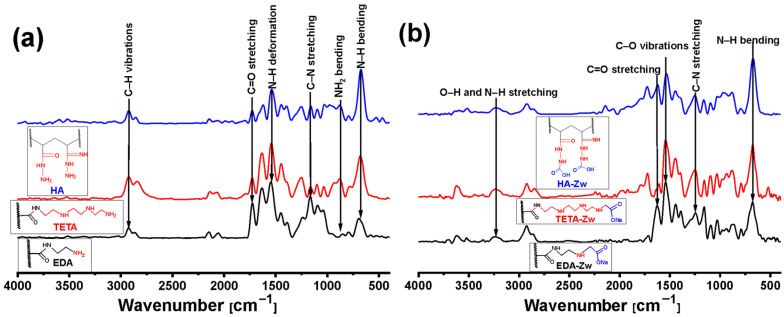
FTIR-ATR spectra of cationic IExRs (**a**) and the subsequent zwitterionic functionalization (**b**).

The FTIR-ATR spectra provide solid evidence of the chemical structure of the beads, both for the cationic IExRs and the subsequent zwitterionic functionalization (Figure 1).

The FTIR-ATR spectra of the amine-modified ion exchange resins (IEx-EDA, IEx-TETA, and IEx-HH) are displayed in Figure 1a. All samples revealed characteristic absorption bands establishing the successful functionalization of the polymer backbone with primary and secondary amine-containing groups, both of which are important for the future preparation of zwitterionic resins. The bands located near 2900 cm^−1^ in all the spectra represent C-H stretching from the aliphatic groups located within the resin matrix. The IEx-EDA resin exhibited a band in the range of 1350–1450 cm^−1^ characteristic of C–N stretching. A moderate band located at approximately 1560 cm^−1^ was assigned to N-H bending, which encompasses primary and secondary amine bonds. The absence of a carbonyl-related signal provides further relevant evidence that functionalization predominantly occurred via amine grafting to the resin surface with ethylenediamine moieties introduced. The IEx-TETA resin (Figure 1a) demonstrates similar, but more intense (and broad) absorptions in the N-H stretching range, indicating an increased amount of amine groups due to triethylenetetramine incorporation. The increase in intensity between both N-H deformation (~1550–1600 cm^−1^) and C–N stretching (~1350–1450 cm^−1^) suggests the presence of multiple amine environments (primary and secondary amines). The increases in amine concentrations would be expected to enhance the reactivity of the resin in further functionalization to zwitterionic structures. In contrast to the previous two IExRs, the IEx-HH resin (Figure 1a) had a moderated absorption band near 1650 cm^−1^, indicative of the C=O stretching vibrations associated with hydrazide or amide groups (–CONHNH_2_). Also, the presence of the C = N group is set in an N–H-rich environment, which weakens a substantial amount of double-bond character through conjugation/resonance; N–H···N interactions would shift the frequency toward ~1690 cm^−1^ (a weak and partially masked band). The detection of additional N–H deformation and C–N stretching bands indicates the effective substitution of these hydrazide groups. These functional groups provide nucleophilic (–NH–) and electrophilic C=O sites, making IEx-HH a unique multifunctional intermediate to develop zwitterionic-type resins where charge is evenly distributed. The incremental change in intensities and positions among the three spectra suggest that more amine substitution and molecular complexity are present. Chemical change such as this can influence the density and functionality of reactive sites available for subsequent sulfonation or carboxylation reactions that are essential for producing zwitterionic-type resins, which display an increase in hydrophilicity and charge neutrality and can potentially be obtained under ion exchange conditions.

The characteristic peaks of Zw confirm the presence of key functional groups associated with improved hydrophilicity, charge neutrality, and antifouling potential, as would be expected from amphoteric surface engineering. Thus, the characteristic bands of IEx-EDA-Zw derived from EDA (Figure 1b) are as follows: the bands characteristic of the COO^−^ group at 1625 cm^−1^ (C=O stretching) and at 1545 cm^−1^ (C–O vibrations), as well as those for the amino groups at 1395 cm^−1^ and 1249 cm^−1^ (symmetric and asymmetric C–N stretching), thus confirming the formation of Zw. The weak band at 3242 cm^−1^, assigned to N–H and O–H stretching, and that at 677 cm^−1^, characteristic of N–H out-of-plane bending, along with the broadness and multiplicity in the region 3600–3200 cm^−1^ can suggest hydrogen bonding, likely due to hydroxyl groups (from Zw) and amine groups (from EDA). Moreover, broad and overlapping bands in the 3000–3600 cm^−1^ region indicates hydrophilicity and hydrogen bonding, consistent with what is expected for zwitterionic surfaces.

For the IEx-TETA-Zw, which is obtained from TETA (Figure 1b), the presence of broad N–H/O–H bands (3237 cm^−1^, 1444 cm^−1^, and 677 cm^−1^), C=O stretching vibrations (1726 cm^−1^), and C–O/C–N peaks (1546 cm^−1^ and 1246 cm^−1^) confirm the IExR chemical structure by providing evidence for the functional groups of both TETA and Zw. Similarly, the IEx-HH-Zw spectrum exhibits all the expected features of both hydrazine hydrate and COOH-containing polymer (IEx-HH-Zw) (Figure 1b). The absorption bands at 3515 cm^−1^ and 3230 cm^−1^ from the HH-Zw spectrum are attributed to the vibration of NH_2_ and O–H groups. Also, the characteristic bands of carboxylic groups are present in the spectrum at 1533 cm^−1^ and 1726 cm^−1^, indicating ester/carboxyl functionalities characteristic of IEx-HH-Zw. The presence of primary amines in Zw is demonstrated by the bands at 2141 cm^−1^ (N–H stretching vibration), 1444 cm^−1^ (N–H bending and C–N stretching vibration), 1095 cm^−1^ (NH_2_ deformation vibration), and 674 cm^−1^, characteristic of N–H out-of-plane bending vibrations. The bands at 1622 cm^−1^ (C = N stretching vibrations) and 1251 cm^−1^ (C–N stretching vibrations) are assigned to the CN group.

**Figure 2:**
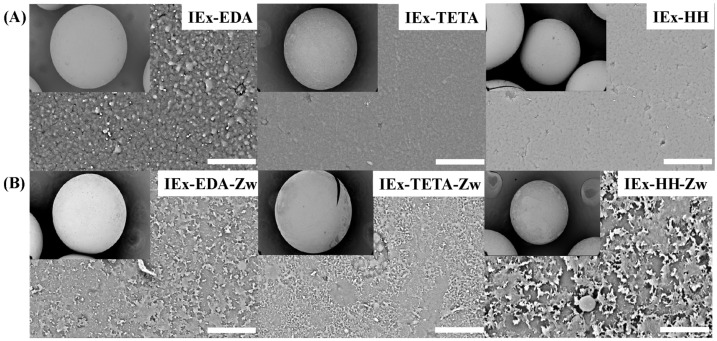
SEM images on the surface of IExRs with weak cationic groups (**A**) and the corresponding zwitterionic resins (**B**) (300 μm scale bar). Inset images show lower magnification SEM images of the corresponding IExRs (5 μm scale bar).

The morphological characterization of IExRs, before and after functionalization with sodium chloroacetate, was performed using SEM (Figure 2).

Initially, the weak cationic IExRs have either a rough and granular (EDA) or a smooth with microcracks surface (TETA and HH). After functionalization, the surface of IEx-EDA-Zw became rougher, and the zwitterionic layers appeared to have formed a loosely packed, possibly hydrated or porous structure; also, the visible fibrous/rod-like feature may suggest polymer aggregates. In the case of IEx-TETA-Zw, the surface shows a heterogeneous structure with polymer clustering; some areas look smoother, while others show distinct domains (indicative of partial or uneven polymer coverage). The surface of IEx-HH-Zw is very porous and highly textured morphology, which suggests substantial surface modification and potentially high polymer loading. The sponge-like architecture may enhance hydrophilicity and antifouling properties. Also, in all IExRs cases, the inset images confirm that the spherical geometry is retained after all chemical modifications. Moreover, the surface morphology changes significantly upon zwitterion grafting, indicating successful surface functionalization. The trend from smooth to increasingly porous surfaces from top to bottom reflects the effect of zwitterionic polymer type and interaction with the weak cationic IExRs base material.

**Figure 3:**
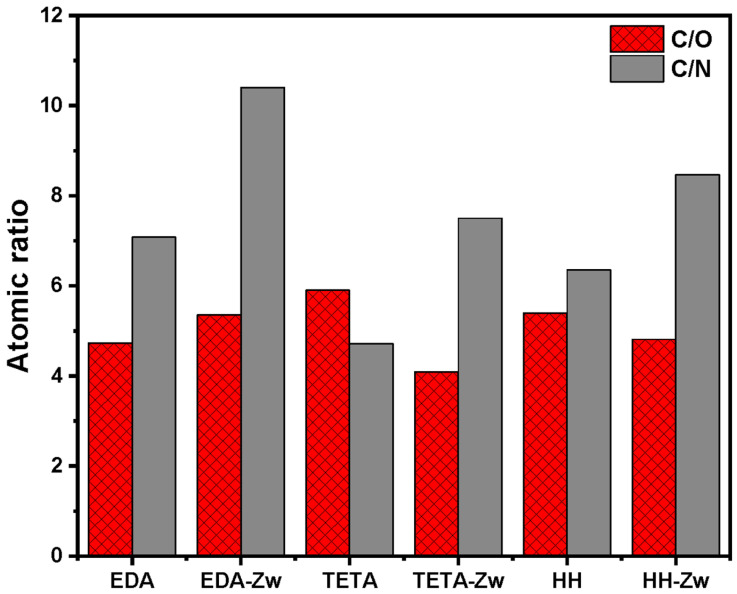
Atomic ratios of C/O and C/N determined by EDAX for cationic IExRs (functionalized with EDA, TETA, or HA) and their zwitterion-functionalized counterparts (EDA-Zw, TETA-Zw, HH-Zw).

Elemental analysis conducted by EDAX was performed to emphasize the zwitterion functionalization of the polymer samples. Thus, the C/N atomic ratio, as shown in Figure 3, increases upon functionalization (e.g., IEx-EDA = 4.7 to IEx-EDA-Zw = 10.4), establishing that the zwitterionic groups were successfully added, and thus lowering the overall N content in the zwitterionic beads. Also, the C/O ratios are decreasing, which indicates that the content of oxygen was increased by the chemical modification, by the new added carboxylic group. Overall, these results quantitatively support the introduction of zwitterion moieties, and follow the trend observed in sorption capacity (i.e., a direct effect between the degree of functionalization and sorption capacity).

Table 1 provides comparative data on weak cationic IExRs and the corresponding Zw resins, specifically focusing on their exchange capacities (acidic and basic), volume weight, and mean particle diameter. IEx-TETA-Zw has the highest basic exchange capacity (6.806 mEq·g^−1^) and moderate acid exchange capacity (2.803 mEq·g^−1^), indicating its strong potential in base exchange processes, making it ideal for capturing anions or working in basic environments.

Moreover, IEx-TETA-Zw has the largest particles (0.611 mm), which may influence column performance (e.g., flow rate, back pressure) in future dynamic sorption experiments, but can be ideal for basic ion exchange processes with high capacity and larger particle size for sorption in batch conditions or in a fluidized bed column (dynamic condition). The least basic exchange is seen in IEx-EDA (2.859 mEq·g^−1^), suggesting less functionality for ion exchange. IEx-EDA-Zw and IEx-HH-Zw show similarly high exchange capacities (E, 4.859 mEq·g^−1^ and 4.828 mEq·g^−1^ for acid exchange capacity, respectively, 3.311 mEq·g^−1^ and 3.212 mEq·g^−1^ for basic exchange capacity), probably due to the rigidity of structure (chain length) compared with IEx-TETA-Zw, which are more flexible (longer chain length). However, the weak cationic IExRs resulted in smaller mean bead diameter (Dm) compared to that of the corresponding zwitterionic IExRs (0.318 vs. 0.509 for IEx-EDA-Zw, 0.422 vs. 0.611 for IEx-TETA-Zw, and 0.235 vs. 0.325 for IEx-HH-Zw). The zwitterionic modifications of cationic IExRs tend to enhance both exchange capacities and particle size, possibly due to increased functional group density or swelling behavior. The cationic resin with HH has the lowest weight volume (Wv = 0.054 g·mL^−1^) and the smallest particle size (Dm = 0.235 mm), indicating that it is very light and porous, and sustained by the higher Dm/Wv ratio. This is beneficial for its high surface area but may result in higher pressure drops in packed columns. TETA has the highest Wv (0.390 g·mL^−1^), suggesting a denser resin, sustained also by SEM measurements (Figure 2). In general, Wv appears to inversely correlate with exchange capacity (mEq·g^−1^) in several cases.

### 3.2. Application of Zwitterionic IExR

**Figure 4:**
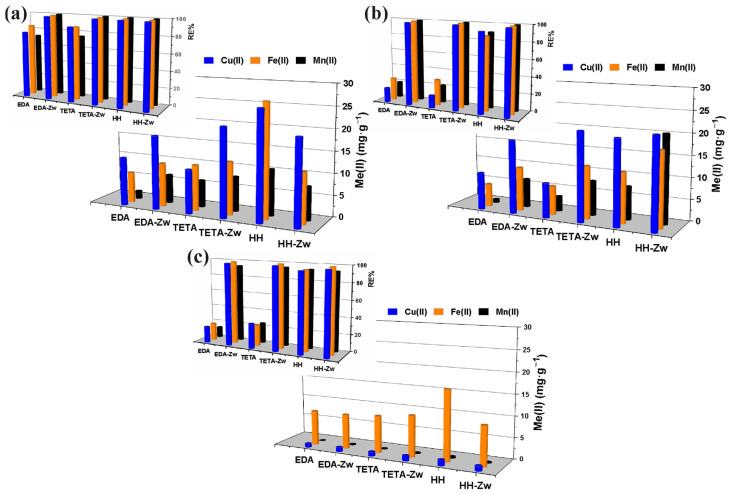
The sorption capacity of the tested IExRs, in (**a**) mono-HMI and (**b**) multi-HMI aqueous solutions, and (**c**) Tarnita-sampled water. Insets of each system’s retention efficiency (RE, %).

The IExRs were evaluated in batch studies as sorbents for several harmful HMIs, utilizing both mono-HMI and multi-HMIs solutions, and also a real water sample from the Tarnita tailing pond, with the findings illustrated in Figure 4. Consequently, in mono-HMI and multi-HMIs solutions, the functionalized IExRs beads with EDA, TETA, and HH exhibited values of SC of between 2 and 27 mg HMIs/g resin, regardless of the tested simulated polluted water (Figure 4a). The AAS results (Figure 4a,b) indicate that both EDA- and TETA-based zwitterionic resins demonstrated the greatest sorption performance across all tested HMIs, irrespective of mono-HMI and multi-HMIs tested solutions. At the same time, IEx-HH-Zw presented a lower SC compared to the starting resin based on HH, but a higher RE (more than 99%).

Additionally, compared with the EDA-based resins, the TETA-functionalized resin and the corresponding zwitterionic one exhibits greater SC regardless of the tested synthetic contaminated solutions (mono- or multi- HMIs), probably due to TETA’s longer sidechains and increased flexibility. Nevertheless, the competitive conditions modified the RE of the cationic (EDA, TETA, and HH) resin, whereas the zwitterionic resin did not cause any notable changes in the SC and RE, comparable with the monocomponent solution. The examined resins were efficient in the sorption of all tested HMIs in both mono- and multi-HMIs systems, with Cu(II) > Fe(II) > Mn(II) as the order, with a small exception in the case of HH-Zw, which had a slightly higher affinity for Fe than Cu in monocomponent solution. When the tests were performed on the Tarnita-sampled water, the EDA- and TETA-based zwitterionic resins and the HH series retained about 93.8% Mn(II), 94.7% Fe(II) and more than 95.5% Cu(II) from real water (Table 2), even though Tarnita water had a significant excess of the Fe(II) ion (154.49 mg·L^−1^) compared to the Cu(II) and Mn(II) (19.91 and 1.95 mg·L^−1^, respectively) content (Figure 4c, Table S2).

**Figure 5:**
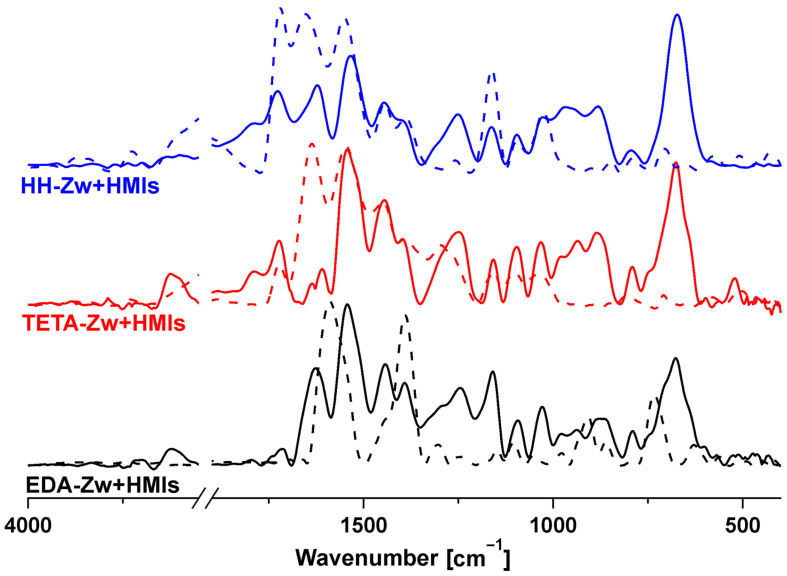
FTIR-ATR analysis before (dash lines) and after (solid line) multi-HMI sorption.

Figure 5 depicts FTIR-ATR spectra of IExRs with EDA-Zw, TETA-Zw, and HH-Zw before and after sorption from the multi-HMIs. The broad absorption band in the region of 3400–3300 cm^−1^, assigned to O-H and N-H stretching vibrations, decreased in intensity and slightly shifted downwards after the adsorption process, suggesting possible hydrogen bonding and/or coordination interactions with metal ions in solution. The band present in the 1630–1650 cm^−1^ region ascribed to C=O stretching from carboxyl or amide moieties exhibited a downward shift and clearly lost intensity, which adds to the strength of the hypothesis that these functional groups participated in metal coordination processes. Additional support for nitrogen- and oxygen-donor sites as participants to Me(II) complexation is suggested by the relatively small shifts observed in the 1100–1000 cm^−1^ region (C-N/C-O motions). Collectively, these spectral changes support the conclusion that Me(II) interacts with the bound functional groups present on the surface through coordination or electrostatic attraction effects, as in Ref. [16], to reinforce the proposed chelation–ion exchange adsorption mechanism that may occur in a mixed component system.

In this study, the HMIs content of synthetically contaminated waters and that collected from the Tarnita region are evaluated and contrasted to the HMIs permissible values as maximum limits for surface waters, as determined by the world and European specialized organisms (Table S2) [17,18]. In this respect, the IEx-EDA-Zw, IEx-TETA-Zw, IEx-HH, and IEx-HH-Zw resins successfully cleaned contaminated water up to below the admissible limit (Table 2). This shows the possible use of these IExRs, with a high crosslinking degree, in surface water treatment, with similar results as in our previous study, where 3% crosslinking degree IExRs based on EDA and TETA were tested [16] (Table S2).

In order to gain information on the optimum conditions for the sorption of tested HMIs from real wastewaters by the IEx resins prepared in this work, batch sorption experiments were performed to investigate the isotherms, kinetics, and thermodynamics of the sorption process using simulated aqueous solutions of mixtures of the four HMIs. This research opted for a multicomponent system to better depict the actual polluted water composition. Real wastewater typically consists of a complicated mixture of organic and inorganic contaminants rather than a single solute. Thus, by using a multicomponent system, the model is able to capture the competition, sorption, and transport of solutes as would typically be expected under realistic environmental conditions. A multicomponent system provides a better assessment of the material’s performance for practical water treatment applications. The experimental isotherm and kinetic sorption data were fitted with several well-known models (Table S1).

**Figure 6:**
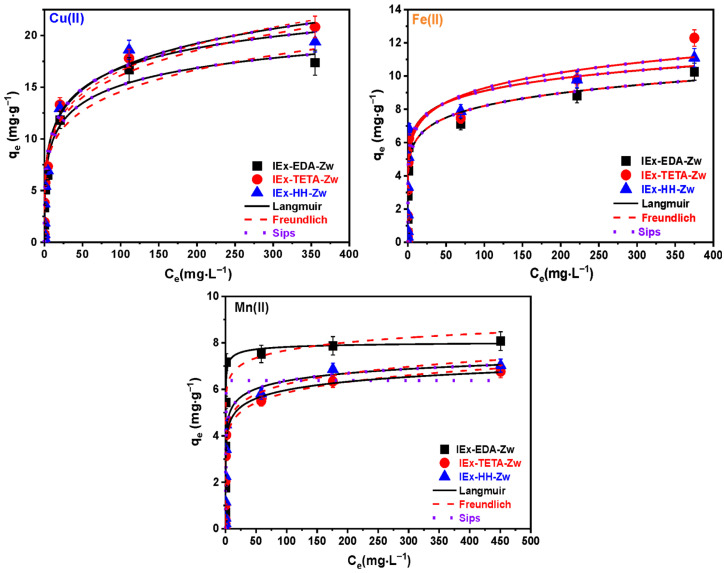
Equilibrium data for the sorption HMIs from multicomponent solutions using IEx-EDA-Zw, IEx-TETA-Zw, and IEx-HH-Zw resins.

The experimental equilibrium data showed that the Zw has a high affinity for the tested HMIs, as demonstrated by the “Class L” isotherms shape [38] obtained for all systems (Figure 6).

Once again, it is observed that the IEx-TETA-Zw and IEx-EDA-Zw resins had the highest and the lowest, respectively, sorption capacities for all HMIs. In this study, the experimental equilibrium results have been fitted with three well-known equations, namely the isotherms Langmuir, Freundlich, and Sips (Table S1). The parameters obtained by modeling the experimental data with the three isotherm models are presented in Table 3. The Langmuir model was used to characterize monolayer adsorption on a homogeneous surface with identical active sites, whereas the Freundlich model depicted adsorption on heterogeneous surfaces with different affinities. The Sips model was selected to account for adsorption on heterogeneous surfaces, since it is a combination of the Langmuir and Freundlich models. The use of these models allows for a suitable interpretation of the process of adsorption in multicomponent systems, taking into account surface heterogeneity and potential interactions of adsorbed species. The suitability of a certain isotherm model to fit the experimental results was evaluated by the *R^2^* correlation coefficient, i.e., the higher the *R^2^* value, the better the model described the equilibrium data. As it is seen, the Sips equation could not be fitted for the Mn(II) sorption on IEx-EDA-Zw resin, probably because this metal ion is more prone to transformation into metal oxides. Nevertheless, the Sips isotherm was the most appropriate for describing the experimental data, as demonstrated by the highest *R^2^* values, with the exception of Mn(II) sorption on the IEx-TETA-Zw and the IEx-HH-Zw resins, for which the Freundlich and Langmuir models were more suitable. The favorable adsorption of HMIs on the investigated resins is also supported by the values of the *1/n* parameter in the Sips model, which were between 0.01 and 0.67. Also, the high and consistent values of *R*^2^ (0.93–0.98) and relatively good agreement of *q*_*m*_ and *K* values indicate that these models sufficiently represent the equilibrium adsorption properties of the tested resins.

**Figure 7:**
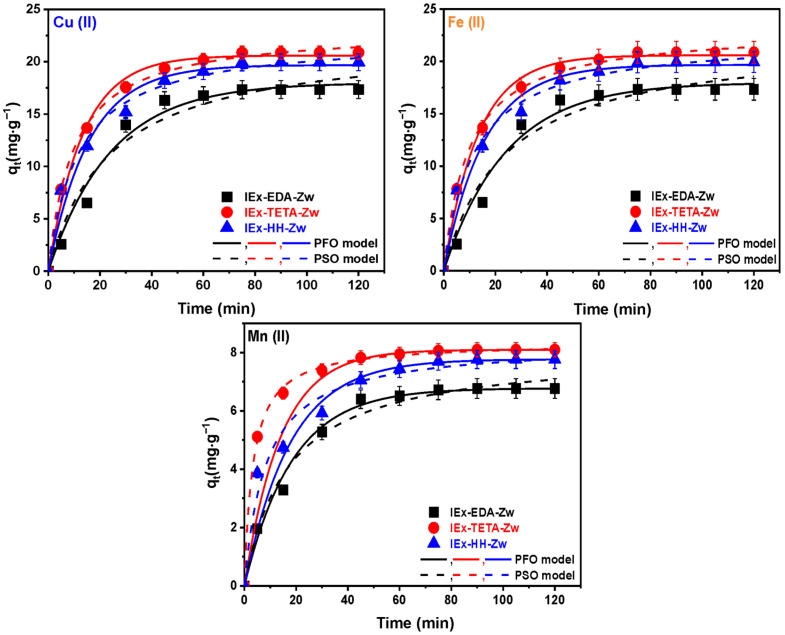
Sorption data of HMIs (multicomponent solution) on zwitterionic resins fitted by PFO and PSO kinetic models.

The effect of contact time on the sorption of Cu(II), Fe(II), and Mn(II) by the IEx-EDA-Zw, IEx-TETA-Zw, and IEx-HH-Zw resins is shown in Figure 7. As can be seen, the maximum sorption capacity is reached in less than three hours for all resins.

To gain information about the sorption mechanism of HMIs on the resins, the experimental data has been fitted with the pseudo-first-order (PFO) and pseudo-second-order (PSO) kinetic models. The kinetic parameters presented in Table 4 show that the sorption of HMIs on the IEx-EDA-Zw resin was better fitted by the PFO model (i.e., physical sorption), whereas PSO was more suitable (i.e., chemical sorption) for the IEx-HH-Zw resin, as reflected by the higher *R^2^* and lower *χ^2^* correlation coefficients. Conversely, for the IEx-TETA-Zw resin, the *R^2^* and *χ^2^* correlation coefficients indicate physical adsorption in the case of Cu(II) and chemical sorption for Fe(II) and Mn(II).

**Figure 8:**
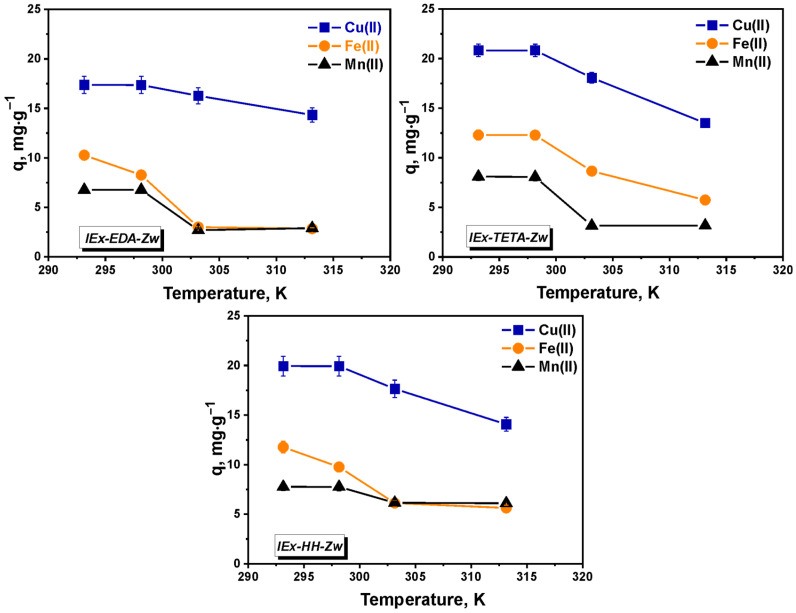
Effect of temperature on the maximum sorption capacity of multicomponent Cu(II), Fe(II), and Mn(II) solutions by zwitterionic resins.

The investigations on the influence of temperature on the sorption processes are important to acquire information on the spontaneity and feasibility of sorption. This can be achieved by assessing parameters such as the standard enthalpy (ΔH°), the standard entropy (ΔS°), and the Gibbs free energy (ΔG°). Figure 8 presents the plots of sorbed amount of HMIs (*q*) versus temperature. As can be seen, an insignificant variation in *q* was determined by increasing the temperature from 20 to 25 °C. Nevertheless, the sorption capacities of the resins decreased for all the tested HMIs by further increasing the temperature up to 40 °C.

The linear regressions of *ln K_d_* vs. *1/T* for the investigated systems (Figure S1) allowed for the determination of standard enthalpy (ΔH°) and standard entropy (ΔS°) using Equation (13), Table S1. The Gibbs free energy (ΔG°) was also calculated using Equation (14), Table S1. The obtained thermodynamic parameters are presented in Table 5. The thermodynamic parameters determined in the present study indicate a spontaneous (negative ΔG°), exothermic (negative ΔH°), and decreased disorder (negative ΔS°) sorption of metal ions onto IExRs, which is substantially different from observations in the literature that commonly report metal ion sorption kinetics as being endothermic with positive values of entropy. As evidence, Moradi et al. [39] have shown that the positive ΔH° and ΔS° values measured for the adsorption of Cu(II) and other heavy metals onto polymeric resins are related to the release of hydration water molecules and the increase in disorder at the solid–liquid interface. The phosphate-cellulose fiber biosorbents displayed endothermic sorption kinetic processes, as these processes are entropy-driven [40]. The negative ΔH° and ΔS° seem to conform more to the study of synthetic resins in which sorption contributes to the ordering of the sorption mechanism due to strong binding interactions observed, for example, with Amberlite IRA-400 with a synthetic resin [41]. Within this study, it could be concluded that the relatively high negative ΔH° values (−215 to −377 kJ·mol^−1^) indicate that the involved interactions must be strong and more than likely include chelation or multi-point binding sites that limit the structure of the sorbed ions, and contribute to the loss of entropy during the sorption process. The loss of entropy during the adsorption process is a result of the immobilization of metal ions on the resin and the potential reorganization of solvent molecules to develop a more ordered system that overcomes any entropy gained by the displacement of water molecules with the chelating of metal ions. These findings indicate a different mechanism of adsorption than most described adsorption processes, based on their endothermicity and thermodynamically driven by increases in entropy, and demonstrate the specific physicochemical character of the studied resin systems.

**Figure 9:**
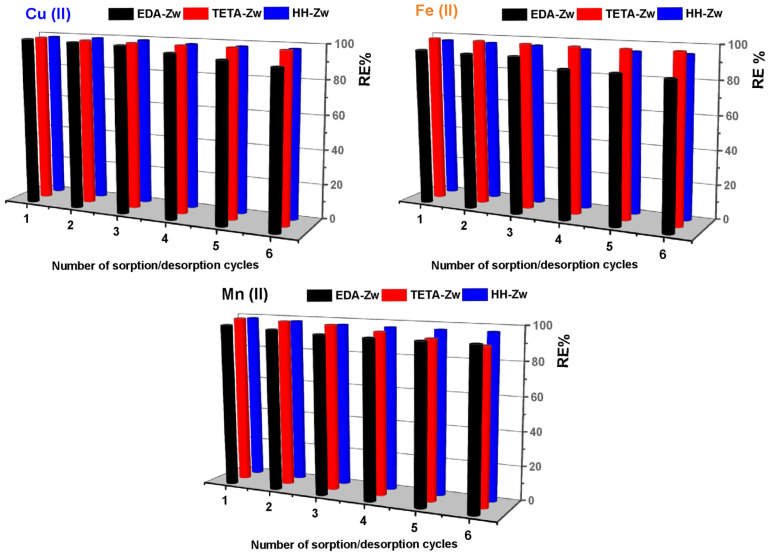
The zwitterionic IExRs retention efficiency (RE, %), tested for multi-HMIs solutions over six cycles of sorption/desorption.

For economic viability, sorbents must quantitatively desorb stored solutes and facilitate the regeneration of full active sites. This study evaluated the reutilization of zwitterionic ion exchange resins for the sorption of Cu(II), Fe(II), and Mn(II) during six sorption/desorption cycles (Figure 9). The regeneration efficiency of zwitterionic resins in a multi-HMIs solution after six cycles of sorption/desorption was higher than 90%, as shown in Figure 9 and Figure S2. The Zw showed excellent stability and reusability under the tested conditions, and no evidence of structural damage, discoloration, or cracking was observed after six sorption–desorption cycles, indicating good mechanical integrity of the IExRs. This indicates that the sorbent and its functional groups have not been damaged during any desorption/regeneration cycles. According to these findings, the tested IEx resins with an 8% crosslinking degree show promise as a material for removing HMIs from synthetic and real polluted water, with similar or even superior performances to the similar resins with a 3% crosslinking degree [16]. These results also show that ion exchange resins can be recycled for a minimum of six sorption/desorption cycles and that the sorption process is reversible.

### 3.3. Wheat Germination Tests

**Figure 10:**
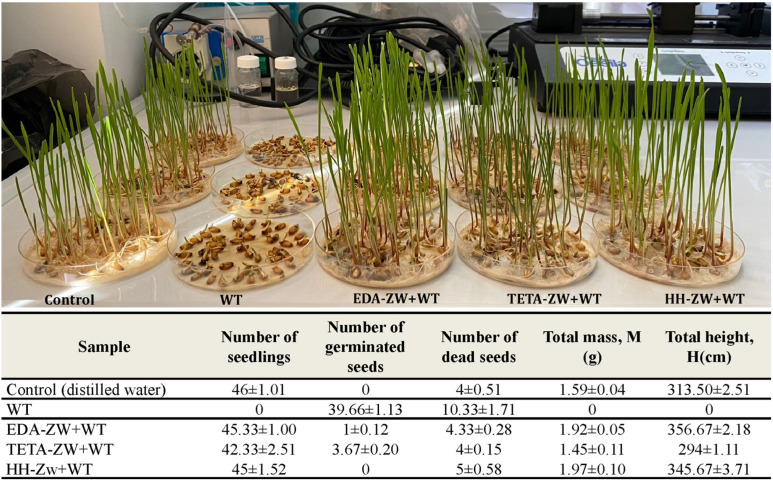
Impact of zwitterionic IExR sorption on the toxicity elimination of water sampled from Tarnita (WT) on wheat germination seeds.

To demonstrate IExRs efficiency in the removing the HMIs from polluted waters, a series of wheat grain germination tests were performed to serve as clear, reliable, and inexpensive techniques acting as a toxicity control tests. As can be seen from Figure 10, the water sampled from Tarnita (WT) fully blocked viable seedling development, whereas the control sample (distilled water) resulted in a total plantlets height of 313.5 cm. The supernatant obtained after IExR HMIs sorption did not stop seeds from germinating or seedlings from growing. Furthermore, after seven days germination, the tested supernatants had a harmless impact on wheat seedlings, with the total mass values closely resembling those of the control (1.92 g for IEx-EDA-Zw, 1.45 g for IEx-TETA-Zw, 1.97 g for IEx-HH-Zw, and 1.59 g for the control). It is worth mentioning that the supernatants obtained after IEx-EDA-Zw and IEx-TETA-Zw HMIs sorption even led to an increase in the H values at 356.7 and 345.7 cm. Given that the Tarnita-tainted water had a hazardous impact on germination, whereas the resultant water after the sorption test was harmless, it can be assumed that the zwitterionic IExRs constitute effective materials for water cleaning.

Many scientific studies have reported high capacities or removal efficiencies for single metal or idealized laboratory solutions, but relatively few studies have reported performance in multicomponent systems or in actual field water, where there is competition from ions and matrix complexity. The Zw tested in the current work experienced removal efficiencies ~94–95% for simultaneously removing three metal ions (Cu(II), Fe(II), and Mn(II)) in a real water matrix, suggesting that it functions robustly under realistic conditions. In contrast, the literature reports on conventional IExRs have demonstrated somewhat less efficiency or varied performance. For example, Dowex 50W was reported to achieve maximum recoveries of approximately 97% of Cu(II), Zn(II), Ni(II), and Cd(II) at pH 8–9 in a single metal system, but was less selective toward Pb(II), which was ~80% in the resin matrix [42]. In another study utilizing Amberlite IR 120 it was reported that at the conditions utilized, Fe from a bis(2 hydroxyethyl) terephthalate (B-HET) solution showed dramatically different efficiencies, e.g., 66% for Fe and~50% for Ni [43]. The selectivity of IExRs is driven by factors that can include ionic radius, charge density, coordination behavior, and functional group affinity. For instance, the order Pb(II) > Cd(II) > Cu(II) was found in competitive systems in one study that utilized an amino bio resin [44]. In our research study, the order Cu(II) > Fe(II) > Mn(II) is in concordance with our zwitterionic functional group’s elevated affinity toward the more strongly coordinating or higher charge-density ion (Cu(II)) and could support some of the advantages that are attributed to its functionalization over basal cation exchange, as already demonstrated in our previous study, where EDA- and TETA-based resins with 3% DVB were used in similar conditions [16]. Unmodified or generic cation exchange resins (i.e., sulfonated resins) have weak selectivity or only moderate removal in complex water matrices, as previously demonstrated by Gupta et al. [45] and Katariya [46]. By comparison, the results obtained with our resins suggest that the zwitterionic functional groups could provide both more adsorption capacity/efficiency and more resiliency, under multicomponent HMIs, and in real-water conditions, while other modified generic resins have reported much lower capacities (e.g., capacity ~40 mg/g for Zn(II) on a surfactant–EDTA-modified resin [47]. In our experience, this study has removal efficiencies in the order of >90% in a metered real matrix, which is comparable or even better than the many literature-reported results, especially if one accounts for the complexity.

In summary, although high-performance commercial resins can achieve great removal in simpler systems, the zwitterionic resin tested herein exhibits two main advantages: (1) high removal efficiency, even in a real complex water; and (2) consistency in selectivity order (Cu > Fe > Mn), both in synthetic prepared multicomponent HMIs solutions and in water collected from the Tarnita area.

## 4. Conclusions

This paper presents a detailed look at the synthesis and characterization of IExRs made from acrylic copolymers with 8% crosslinking, along with their application in HMIs removal from synthetic (mono- and multicomponent) and real polluted water (Tarnita water). The structural strength and surface properties of the resin are key for HMI removal abilities, properties confirmed by FTIR-ATR and SEM techniques. SEM imaging revealed the resin’s surface features and texture, giving insights into the surface morphologies, and the FTIR-ATR analysis detected the functional groups, showing that the Zw synthesis was successful. The IEx-EDA-Zw, IEx-TETA-Zw, and IEx-HH-Zw functionalized resins showed effective HMI removal in both simulated and real Tarnita-polluted waters. IEx-TETA-Zw’s structural flexibility likely contributed to it having the highest removal capacity among all the ions studied. Even in competitive conditions, Zw showed high removal rates, with small variation compared to the monocomponent system. The resins managed to remove over 93.8% of Mn(II), Fe(II), and Cu(II) from polluted Tarnita water, proving their potential for cleaning water. The results show that the Zw resins remained effective and stable after for more than six sorption/desorption cycles, showing good reusability and permanence and possible use for sustainable water treatment options and lower costs. The removal of heavy metal ions with the resins was achieved from both natural and synthetic polluted waters in mono- and multicomponent systems, demonstrating practical applicability and compatibility with existing water treatment systems. The findings on wheat germination show that Zw effectively removes harmful HMIs from Tarnita-polluted water, as evidenced by the improved germination and growth of wheat seeds. Unlike the untreated Tarnita water, which completely inhibited seedling development, the supernatants obtained after HMIs sorption were nontoxic and supported normal plant growth. Notably, the zwitterionic resins maintained their performance under competitive conditions and showed no phytotoxic effects after treatment, confirming their potential for safe and efficient application in environmental water decontamination.

However, these findings are derived solely from laboratory-scale batch studies and should therefore provide only preliminary evidence of larger scale and environmental benefits. Further pilot-scale or field-scale studies, including life-cycle assessment and long-term stability studies, are required to demonstrate whether the Zw resins provide environmental benefits and represent viable alternatives for water treatment technologies.

## Figures and Tables

**Table 1:** The characteristics of the weak cationic IExR and their functionalized Zw.

IEx Resins	E(Acid)		E(Basic)		Wv	Dm
	mEq·mL^−1^	mEq·g^−1^	mEq·mL^−1^	mEq·g^−1^	g·mL^−1^	mm
**IEx-EDA**			1.154	2.859	0.295	0.318
**IEx-EDA-Zw**	4.669	4.859	0.959	3.311	0.188	0.509
**IEx-TETA**			2.067	5.650	0.390	0.422
**IEx-TETA-Zw**	1.646	2.803	1.334	6.806	0.157	0.611
**IEx-HH**			0.225	1.915	0.054	0.235
**IEx-HH-Zw**	1.392	4.828	2.153	3.212	0.164	0.325

**Table 2:** Concentration of HMIs after resin sorption from mono-HMI (MoHMI) and multi-HMIs (MuHMI) synthetic aqueous solutions, and from water sampled from Tarnita (WT).

Sample	Cu(II) (mg·L^−1^)			Fe(II) (mg·L^−1^)			Mn(II) (mg·L^−1^)		
	MoHMI	MuHMI	WT	MoHMI	MuHMI	WT	MoHMI	MuHMI	WT
**IEx-EDA**	2.36 ± 0.20	4.58 ± 0.50	14.92 ± 0.90	7.37 ± 0.60	10.62 ± 1.10	110.53 ± 5.30	3.44 ± 0.50	15.16 ± 1.00	1.71 ± 0.03
**IEx-EDA-Zw**	0.23 ± 0.01	0.85 ± 0.01	0.12 ± 0.01	0.13 ± 0.01	0.16 ± 0.01	0.18 ± 0.02	0.12 ± 0.01	0.28 ± 0.02	0.13 ± 0.03
**IEx-TETA**	6.78 ± 0.10	9.58 ± 0.20	12.89 ± 0.50	3.56 ± 0.20	12.09 ± 0.60	116.85 ± 1.30	3.91 ± 0.20	16.65 ± 0.70	35.2 ± 0.80
**IEx-TETA-Zw**	0.15 ± 0.01	0.5 ± 0.03	0.11 ± 0.04	0.10 ± 0.02	0.12 ± 0.04	0.16 ± 0.02	0.1 ± 0.01	0.24 ± 0.01	0.1 ± 0.03
**IEx-HH**	0.36 ± 0.03	1.12 ± 0.03	1.09 ± 0.30	0.11 ± 0.03	0.21 ± 0.03	0.19 ± 0.03	0.17 ± 0.03	0.29 ± 0.03	0.23 ± 0.03
**IEx-HH-Zw**	0.37 ± 0.01	0.84 ± 0.03	0.29 ± 0.04	0.12 ± 0.06	0.19 ± 0.07	0.15 ± 0.09	0.22 ± 0.06	0.27 ± 0.02	0.18 ± 0.05

**Table 3:** Langmuir, Freundlich, and Sips isotherm models parameters for the sorption of tested zwitterionic resins for Cu(II), Fe(II), and Mn(II).

IEx Resins	HMIs	Isotherm Model									
		Langmuir			Freundlich			Sips			
		q_m,_ (mg·g^−1^)	K_L,_ (L·mg^−1^)	R^2^	K_F_, (L·g^−1^)	1·n^−1^	R^2^	q_m_, (mg·g^−1^)	a_S_	1·n^−1^	R^2^
**IEx-EDA-Zw**	**Cu(II)**	28.54 ± 1.10	0.23	0.9343	5.88 ± 0.80	0.20	0.9357	28.48 ± 2.10	0.35	0.23	0.96601
	**Fe(II)**	523.74 ± 10.10	0.01	0.9169	4.51 ± 0.60	0.13	0.9377	498.97 ± 5.40	0.13	0.01	0.9556
	**Mn(II)**	8.16 ± 1.20	3.64	0.9166	5.75 ± 0.70	0.06	0.7750	-	-	-	-
**IEx-TETA-Zw**	**Cu(II)**	56.99 ± 0.80	0.12	0.9612	6.44 ± 0.40	0.21	0.9679	**56.77** ± 1.10	**0.27**	**0.12**	**0.9748**
	**Fe(II)**	2297.14 ± 20.10	0.02	0.8603	4.89 ± 0.20	0.14	0.8954	**2083.85** ± 8.10	**0.14**	**0.01**	**0.9398**
	**Mn(II)**	10.89 ± 0.50	0.50	0.9841	**3.53** ± 0.04	**0.11**	**0.9796**	10.89 ± 0.60	0.19	0.50	0.9703
**IEx-HH-Zw**	**Cu(II)**	31.80 ± 0.10	0.22	0.9300	6.35 ± 0.40	0.21	0.9330	**31.72** ± 0.10	**0.36**	**0.22**	**0.9638**
	**Fe(II)**	204.13 ± 0.10	0.03	0.8858	5.33 ± 0.10	0.12	0.9144	**197.83** ± 1.10	**0.12**	**0.03**	**0.9470**
	**Mn(II)**	**9.99** ± 0.10	**0.66**	**0.9714**	3.86 ± 0.20	0.10	0.9644	9.99 ± 0.10	0.21	0.67	0.9637

**Table 4:** Kinetic parameters for Cu(II), Fe(II), and Mn(II) sorption by zwitterionic resins.

	Parameter	IEx-EDA-Zw			IEx-TETA-Zw			IEx-HH-Zw		
		Cu(II)	Fe(II)	Mn(II)	Cu(II)	Fe(II)	Mn(II)	Cu(II)	Fe(II)	Mn(II)
Exp.	q_e,exp,_ (mg·g^−1^)	17.40	10.27	6.78	20.84	12.30	8.12	19.94	11.77	7.76
PFO	q_e,cal,_ (mg·g^−1^)	17.98 ± 0.80	10.71 ± 0.50	6.78 ± 0.10	20.59 ± 0.60	12 ± 1.40	8.10 ± 0.70	19.68 ± 1.20	11.30 ± 0.50	7.77 ± 0.70
	k_1_, (min^−1^)	4.16 × 10^−2^	4.23 × 10^−2^	5.45 × 10^−2^	7.51 × 10^−2^	10.73 × 10^−2^	7.09 × 10^−2^	6.33 × 10^−2^	16.38 × 10^−2^	5.61 × 10^−2^
	R^2^	**0.9831**	**0.9661**	**0.9918**	**0.9925**	0.9824	0.8456	0.9792	0.9565	0.9284
	χ^2^	0.89	0.65	0.06	0.42	0.29	1.13	1.07	0.67	0.52
PSO	q_e,cal,_ (mg·g^−1^)	13.95 ± 2.10	13.24 ± 1.10	8.06 ± 0.60	18.01 ± 3.30	13.14 ± 0.50	8.35 ± 0.20	22.31 ± 0.90	12.09 ± 0.30	8.32 ± 0.10
	k^2^, (min^−1^)	1.48 × 10^−3^	3.22 × 10^−3^	7.48 × 10^−3^	1.88 × 10^−3^	1.15 × 10^−3^	2.07 × 10^−3^	3.88 × 10^−3^	3.35 × 10^−3^	3.08 × 10^−3^
	R^2^	0.4175	0.9438	0.986	0.643	**0.9985**	**0.9985**	**0.9915**	**0.9885**	**0.9765**
	χ^2^	30.51	1.08	0.09	20.26	0.03	0.01	0.4	0.18	0.17

**Table 5:** Thermodynamic parameters for HMIs sorption by zwitterionic resins.

IEx Resins	HMIs	ΔH°, (kJ·mol^−1^)	ΔS°, (kJ·mol^−1^·K^−1^)	ΔG°, (kJ·mol^−1^)			
				293.15 K	298.15 K	303.15 K	313.15 K
**IEx-EDA-Zw**	Cu(II)	−217.981	−0.709	−9.505	−5.963	−2.420	−0.664
	Fe(II)	−300.271	−0.100	−5.816	−3.793	−2.229	−1.274
	Mn(II)	−237.301	−0.791	−5.350	−4.394	−3.562	−1.474
**IEx-TETA-Zw**	Cu(II)	−364.445	−1.189	−15.837	−9.891	−3.945	−0.947
	Fe(II)	−334.404	−1.099	−12.307	−6.813	−1.319	−0.468
	Mn(II)	−238.406	−0.795	−5.395	−1.420	−0.754	−0.350
**IEx-HH-Zw**	Cu(II)	−377.505	−1.231	−16.662	−10.507	−4.353	−2.956
	Fe(II)	−232.049	−0.775	−4.811	−2.936	−1.940	−0.692
	Mn(II)	−215.410	−0.706	−8.388	−4.857	−1.326	−0.737

## Data Availability

The original contributions presented in this study are included in the article or Supplementary Materials. Further inquiries can be directed to the corresponding author.

## Supplementary Materials

The following supporting information can be downloaded at https://www.mdpi.com/article/10.3390/polym17233173/s1, Table S1: Mathematical equations used in this study; Table S2: Characteristics of initial mono-HMI (MoHMI) and multi-HMIs (MuHMI) synthetic aqueous solutions, and of water sampled from Tarnita (WT); Figure S1: Plot *ln K_d_* as a function of *1/T* for the sorption of Cu(II), Fe(II), and Mn(II) ions sorption on IEx-EDA-Zw, IEx-TETA-Zw and IEx-HH-Zw resins; Figure S2. Successive HMIs sorption/desorption cycles (multicomponent HMIs).
