# Supplementary material for: Heavy Metals Ions Removal from Local Tarnita Aquatic Streams by Reusable Zwitterionic Acrylic Ion Exchange Resins
Source: Polymers (Basel). 2025 Nov 28;17(23):3173. doi: 10.3390/polym17233173 (PMC12693817; doi:10.3390/polym17233173)
Supplement: Supplementary file 1 [file polymers-17-03173-s001.zip › polymers-3976538-supplementary.pdf]

SUPPORTING INFORMATION

Heavy Metals Ions Removal from Local Tarnita Aquatic Streams by Reusable Zwitterionic Acrylic Ion Exchange Resins

Marcela Mihai, Alina-Petronela Moraru, Ramona Ciobanu, Florin Bucatariu, Marius-Mihai Zaharia*

Petru Poni Institute of Macromolecular Chemistry, 41A Grigore Ghica Voda Alley, 700487 Iasi, Romania

Table S1. Mathematical equations used in this study.

Eq. No	Equation name	Formula	Terms meaning
1.	*Volume exchange capacities of weak base*	$E_{V-WB}(mEq/mL) = \frac{V_{HCl} \cdot f_{HCl} - V_{NaOH} \cdot f_{NaOH}}{V_{IEx}}$	V_{HCl} *(mL)* = volume of hydrochloric acid<br>V_{NaOH} *(mL)* = volume of sodium hydroxide<br>V_{IEx}*(mL)* = volume of ion exchange resin<br>f_{HCl} = factor for HCl<br>f_{NaOH} = factor for NaOH
2.	*Volume exchange capacities of weak acid*	$E_{V-WA}(mEq/mL) = \frac{V_{NaOH} \cdot f_{NaOH} - 5 \cdot V_{HCl} \cdot f_{HCl}}{V_{IEx}}$	V_{HCl} *(mL)* = volume of hydrochloric acid<br>V_{NaOH} *(mL)* = volume of sodium hydroxide<br>V_{IEx}*(mL)* = volume of ion exchange resin<br>f_{HCl} = factor for HCl<br>f_{NaOH} = factor for NaOH<br>*5* = ratio between the total volume of the titration solution and the volume of the sample taken by titration.
3.	*Weight exchange capacities of weak base*	$E_{w-WB}(mEq/g) = \frac{V_{HCl} \cdot f_{HCl} - V_{NaOH} \cdot f_{NaOH}}{W_V}$	V_{HCl} *(mL)* = volume of hydrochloric acid<br>V_{NaOH} *(mL)* = volume of sodium hydroxide<br>W_V*(g/mL)* = volume weight<br>f_{HCl} = factor for HCl<br>f_{NaOH} = factor for NaOH
4.	*Weight exchange capacities of weak acid*	$E_{V-WA}(mEq/g) = \frac{V_{NaOH} \cdot f_{NaOH} - 5 \cdot V_{HCl} \cdot f_{HCl}}{W_V}$	V_{HCl} *(mL)* = volume of hydrochloric acid<br>V_{NaOH} *(mL)* = volume of sodium hydroxide<br>W_V*(g/mL)* = volume weight<br>f_{HCl} = factor for HCl<br>f_{NaOH} = factor for NaOH<br>*5* = ratio between the total volume of the titration solution and the volume of the sample taken by titration.

5.	*Amount of HMI adsorbed at equilibrium*	$SC\ (mg/g) = (C_0 - C_e)V/m$	C_0 (mg/L) = initial concentration of HMI solution;<br>C_e (mg/L) = equilibrium concentration of HMI solution;<br>V (mL) = volume of liquid phase (HMI solution);<br>m (g)= the mass of solid phase.
6.	*Retention efficiency*	$RE = ((C_0 - C_e)/C_o) \times 100$	C_0 (mg/L) = initial concentration of HMI solution;<br>C_e (mg/L) = equilibrium concentration of HMI solution;
7.	*Distribution coefficient*	$K_d(mL/g) = \frac{C_0 - C_e}{C_e}\frac{V}{m}$	K_d = distribution coefficient<br>q_m (mg/g) = the amount of HMIs adsorbed in monolayer;<br>K_L (L/mg) = the Langmuir constant.<br>K_F ($mg^{1-1/n} \cdot L^{1/n} \cdot g^{-1}$) = the Freundlich constant;<br>*1/n* = parameter related to the heterogeneous distribution of active sites on the sorbent surface.<br>a_S = the Sips constant
8.	*Langmuir isotherm model*	$q_e = \frac{q_m \cdot K_L \cdot C_e}{1 + K_L \cdot C_e}$	
9.	*Freundlich isotherm model*	$q_e = K_F \cdot C_e^{1/n}$	
10.	*Sips isotherm model*	$q_e = \frac{q_m \cdot a_S \cdot C_e^{1/n}}{1 + a_S C_e^{1/n}}$	
11.	*PFO kinetic model*	$q_t = q_e(1 - e^{-k_1 t})$	q_e and q_t (mg/g) = the amount of HMI sorbed at equilibrium and at a predefined time;<br>k_1 (min^{-1}) = the rate constant in PFO model;<br>k_2 (g/mg·min) = the rate constant in PSO model.
12.	*PSO kinetic model*	$q_t = \frac{k_2 \cdot q_e^2 \cdot t}{1 + k_2 \cdot q_e \cdot t}$	
13.	*Van't Hoff equation*	$\ln K_d = \frac{\Delta S^o}{R} - \frac{\Delta H^o}{RT}$	ΔS^o = standard enthalpy<br>ΔS^o = standard entropy<br>R = ideal gas constant (8.314 kJ/mol K)<br>T (K) = absolute temperature<br>ΔS^o = Gibbs free energy
14.	*Gibbs free energy equation*	$\Delta G^o = \Delta H^o - T\Delta S^o$	

Table S2. Characteristics of initial mono-HMI (MoHMI) and multi-HMIs (MuHMI) synthetic aqueous solutions, and of water sampled from Tarnita (WT).

Sample	% DVB	Cu(II) (mg·L^{-1})			Fe(II) (mg·L^{-1})			Mn(II) (mg·L^{-1})		
		**MoHMI**	**MuHMI**	**WT**	**MoHMI**	**MuHMI**	**WT**	**MoHMI**	**MuHMI**	**WT**
AML			**2**			**0.2**			**0.3**	
Solution pH		5.16	4.88	3.53	5.21	4.88	3.53	5.43	4.88	3.53
Initial HMI concentration		69.93	70.33	19.91	89.27	87.46	154.59	60.09	65.74	1.95
EDA	8	2.36	4.58	14.92	7.37	10.62	110.53	3.44	15.16	1.71
	3*	1.26	2.12	14.98	1.85	2.98	93.15	0.15	0.24	1.62
EDA-Zw	8	**0.23**	**0.85**	**0.12**	**0.13**	**0.16**	**0.18**	**0.12**	**0.28**	**0.13**
	3*	**0.22**	**0.53**	**0.17**	**0.05**	**0.11**	**0.13**	**0.03**	**0.12**	**0.02**
TETA	8	6.78	9.58	12.89	3.56	12.09	116.85	3.91	16.65	35.2
	3*	3.79	6.93	13.45	2.38	4.24	84.61	0.34	3.68	1.55
TETA-Zw	**8**	**0.15**	**0.5**	**0.11**	**0.10**	**0.12**	**0.16**	**0.10**	**0.24**	**0.10**
	**3***	**0.68**	**1.12**	**0.21**	**0.18**	**0.27**	**0.015**	**0.13**	**0.24**	**0.02**
HH	8	**0.36**	**1.12**	**1.09**	**0.11**	**0.21**	**0.19**	**0.17**	**0.29**	**0.23**
HH-Zw	8	**0.37**	**0.84**	**0.29**	**0.12**	**0.19**	**0.15**	**0.22**	**0.27**	**0.18**

* results from a previuos study [1]

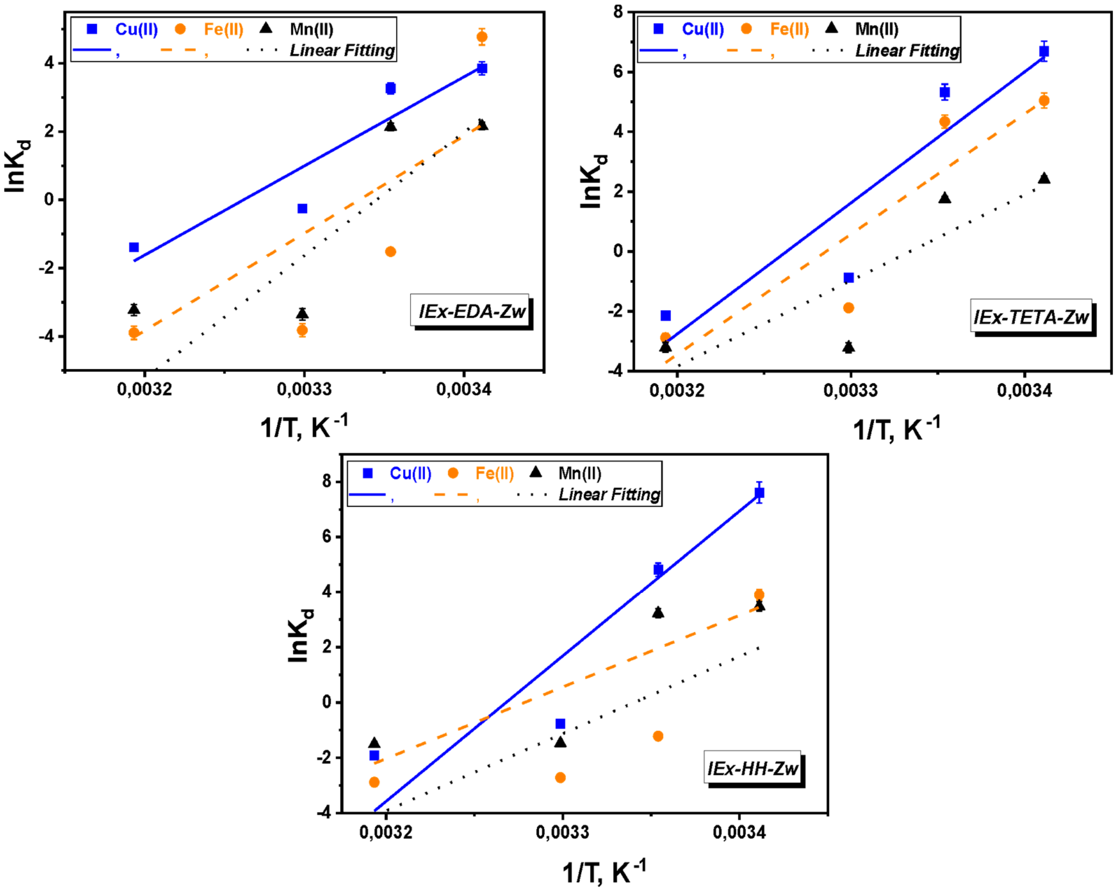

Figure S1. Plot *ln K_d* as a function of *1/T* for the sorption of Cu(II), Fe(II) and Mn(II) ions sorption on IEx-EDA-Zw, IEx-TETA-Zw and IEx-HH-Zw resins.

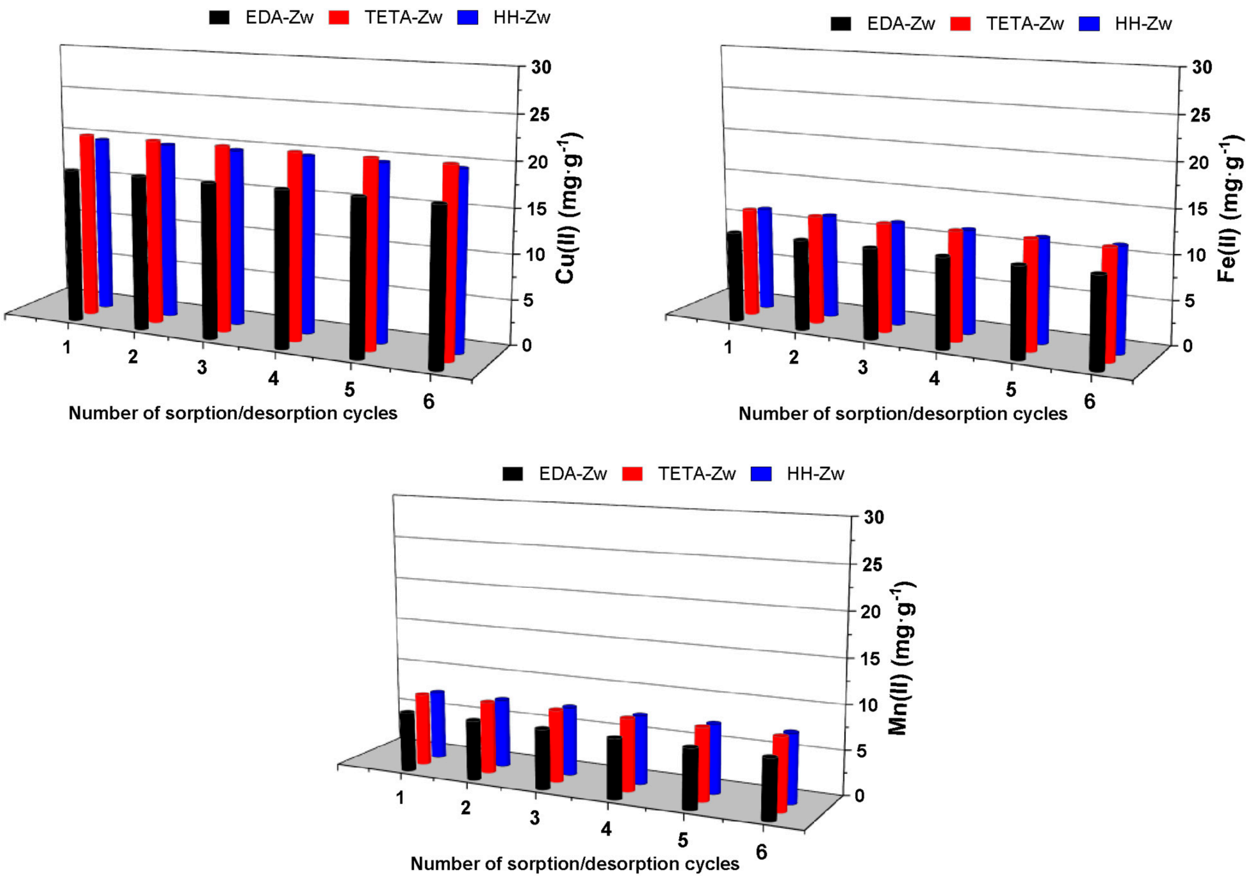

Figure S2. Successive HMIs sorption/desorption cycles (multicomponent HMIs).

References

1. Zaharia, M.M.; Bucatariu, F.; Vasiliu, A.L.; Mihai, M. Stable and reusable acrylic ion-exchangers. From HMIs highly polluted tailing pond to safe and clean water. *Chemosphere* **2022**, *304*, 135383. https://doi.org/10.1016/j.chemosphere.2022.135383
